# Examination of craniofacial morphology in Japanese patients with congenitally missing teeth: a cross-sectional study

**DOI:** 10.1186/s40510-018-0238-9

**Published:** 2018-10-01

**Authors:** Yuki Takahashi, Norihisa Higashihori, Yuko Yasuda, Jun-ichi Takada, Keiji Moriyama

**Affiliations:** Section of Maxillofacial Orthognathics, Department of Maxillofacial/Neck Reconstruction, Graduate School, Tokyo Medical and Dental University, 1-5-45 Yushima, Bunkyo-ku, Tokyo, 113-8549 Japan

**Keywords:** Congenitally missing teeth, Tooth agenesis, Craniofacial morphology, Oligodontia

## Abstract

**Background:**

The purpose of this cross-sectional study was to investigate the effects of congenitally missing teeth on craniofacial morphology and to characterize the features of maxillofacial morphology of oligodontia patients associated with individual skeletal maturity by assessment with the cervical vertebrae maturation (CVM) method.

**Methods:**

A total of 106 non-syndromic Japanese patients with congenitally missing teeth (except for third molars) were selected and categorized into two groups according to the severity of congenitally missing teeth (hypodontia group, 1–5 missing teeth [*n* = 56]; oligodontia group, ≥ 6 missing teeth [*n* = 50]). A control group included orthodontic patients without either skeletal disharmony or congenitally missing teeth (*n* = 63). Subjects in oligodontia and control groups were further categorized into two subgroups on the basis of cervical stage (CS): stage I (CS2 or 3; *n* = 27 and *n* = 31, respectively) and stage II (CS4 or above; *n* = 23 and *n* = 32, respectively). Lateral cephalograms were analyzed by using eight angular and eight linear measurements. Z-scores were formulated on the basis of age and sex and were matched to the Japanese norm. Tukey tests and *t* tests were performed.

**Results:**

Compared with the control group, the hypodontia group had significantly smaller U1 to FH plane angle and A-B plane angle; U1-L1 was significantly larger. The oligodontia group had significantly smaller ANS-Me, L1 to mandibular plane angle, and Ptm-A; U1-L1 was significantly larger. At stage I, the oligodontia group had significantly smaller ANS-Me, gonial angle, and ANS-U1. At stage II, the oligodontia group had significantly smaller U1 to FH plane angle, L1 to mandibular plane angle, Ptm-A, and Go-Pog; it also had significantly larger U1-L1.

**Conclusions:**

The present study suggested that skeletal patterns differ along with the number of congenitally missing teeth and that, in oligodontia patients, skeletal patterns differ before and after growth peak. It is important to consider the skeletal characteristics of tooth agenesis patients when designing a treatment plan.

## Background

Congenitally missing teeth is one of the most common anomalies observed in the maxillofacial region [1–6]. There are two main classifications of congenitally missing teeth: the syndromic type, phenotypically presenting as a syndrome of congenital disorders such as ectodermal dysplasia, and the non-syndromic type, which is simply a disorder characterized by multiple missing teeth [7–9]. The congenital absence of 1 to 5 teeth (excluding third molars) has been described as “hypodontia” by Schalk-van der Weide et al. [10], while the absence of > 6 teeth is known as “oligodontia.” “Anodontia,” a very rare condition, is the absence of all teeth [11]. In Japanese patients, the most frequently missing type of tooth in hypodontia patients was lower second premolars (25.9%), followed by lower lateral incisors (19.4%) and upper lateral incisors (17.1%). In oligodontia patients, lower second premolars (88.2%) were the most frequently missing type of tooth, followed by upper second premolars (87.3%) and upper first premolars (63.7%) [12].

It has been suggested that patients with congenitally missing teeth have specific characteristics of craniofacial morphology and growth patterns. Most authors have found that patients with congenitally missing teeth had a shorter maxilla, a more prognathic mandible, a smaller mandibular plane angle, and greater retroclination of the maxillary and mandibular incisors [2, 13–17], and these characteristics were reported to be affected by the severity of congenitally missing teeth [2, 7, 16, 18–21]. According to Ben-Bassat and Brin [2], these characteristics are probably caused by underdevelopment of the apical base due to the absence of tooth buds. Furthermore, Ogaard and Krogstad [16] reported that the typical dentofacial structure in individuals with severe congenitally missing teeth is due to dental and functional compensation rather than an altered growth pattern. Comparison between samples divided into two categories (5–12 missing teeth and 13–21 missing teeth) [18)], or three categories (2–5 missing teeth, 6–9 missing teeth, and ≥ 10 missing teeth) [16] revealed a more retrognathic maxilla, decrease in the mandibular plane angle, which was caused by reduced occlusal support, resulted in class III malocclusions in the more severely affected congenitally missing teeth. Their findings suggest that patients with severe congenitally missing teeth had unique dental and skeletal patterns, and the severity of congenitally missing teeth is an important factor in craniofacial morphology.

Because the prevalence of non-syndromic oligodontia is ≤ 1% [22], obtaining adequate sample sizes to study is difficult. Moreover, it appears that hypodontia has little effect on general growth patterns according to a longitudinal study by Roald et al. [14]; thus, previous studies examining craniofacial morphology often evaluated subjects with wide age ranges. However, according to alveolar bone development, along with the eruption of permanent teeth [23], it may be speculated that skeletal patterns differ before and after growth, especially in patients with severe congenitally missing teeth. Mogi et al. [24] evaluated craniofacial morphology in growing patients with oligodontia who were limited in dental age range, from III A to III C. They found that growing patients with oligodontia had a shorter anterior lower facial height and a tendency for skeletal class III relationship, compared with Japanese norms. Bondarets et al. [7] also classified patients with oligodontia, based on dental age. They found that patients had a shorter posterior facial height in the mixed dentition, but no significant differences in the permanent dentition, compared with the control group. However, it is difficult to evaluate patients with oligodontia according to dental age because dental age is based on the timing of eruption of permanent teeth [25]. Thus, alternative, yet objective, evaluation methods are necessary for patients with oligodontia. The cervical vertebral maturation (CVM) method is a reliable indicator of individual skeletal maturity and has been used to detect peaks in mandibular growth based on an analysis of the second through fourth cervical vertebrae in a single cephalogram, a type of radiograph routinely available for orthodontic diagnosis. Six maturational stages of these three cervical vertebrae can be determined based on the morphology of their bodies. Cervical stage (CS) 1 and CS2 are prepeak stages, the peak in mandibular growth occurs between CS3 and CS4, and CS6 is recorded at least 2 years after the peak [26–28].

In the present study, we assessed two outcomes. First, we investigated the effect of congenitally missing teeth on craniofacial morphology by classifying patients with congenitally missing teeth, in accordance with the definition by Schalk-van der Weide et al. [10], into a hypodontia group (1–5 missing teeth) and an oligodontia group (≥ 6 missing teeth). Second, subjects in the oligodontia group were further categorized into two subgroups on the basis of cervical stage (CS)—stage I (CS2 or 3) and stage II (CS4 or above)—to characterize features of the maxillofacial morphology of oligodontia patients associated with individual skeletal maturity.

## Methods

A total of 106 non-syndromic Japanese patients with congenitally missing teeth (except for third molars), who were registered at Tokyo Medical and Dental University Dental Hospital in Tokyo, Japan, were enrolled in the study. This was a retrospective hospital-based study. The patients were categorized into two groups on the basis of the severity of congenitally missing teeth: a hypodontia group (1–5 missing teeth) consisting of 56 patients (41 female, 15 male; mean age 17.7 ± 7.8 years), an oligodontia group (≥ 6 missing teeth) consisting of 50 patients (26 female, 24 male; mean age 15.5 ± 6.3 years), and a control group, which included orthodontic patients without either skeletal disharmony or congenitally missing teeth. The distribution of patients, according to the number of congenitally missing teeth in each group, is shown in Table 1. The control group consisted of 63 patients (34 female, 29 male; mean age 17.4 ± 7.6 years). Subjects in the oligodontia and control groups were further categorized into two subgroups according to CS as described by McNamara et al. [28]: stage I (CS2 or 3; *n* = 27 and *n* = 31, respectively) and stage II (CS4 or above; *n* = 23 and *n* = 32, respectively). Dental casts, panoramic radiographs, and lateral cephalometric radiographs (FUFIX FCR7000; Fuji Film, Tokyo, Japan) acquired at the first examination were used as study materials. A diagnosis of congenitally missing teeth was based on clinical and radiographic findings and by eliminating those attributable to injury or caries using a questionnaire form and clinical interviews. In younger children, verification of the number of congenitally missing teeth was based on initial and follow-up treatment records [2]. Lateral cephalometric radiographs of all individuals were acquired at the first visit, and patients’ skeletal characteristics were evaluated. Patients with any associated syndrome, cleft lip/palate, or a previous history of orthodontic treatment were excluded. All procedures in this study were approved by the Ethics Committee of Tokyo Medical and Dental University (no. 419) and complied with the Code of Ethics of the World Medical Association (Declaration of Helsinki). Procedures were fully explained to all participants, who each provided written informed consent before the study. A power analysis was conducted by using G*Power Version 3.1 [29] to justify the sample size with effect size ƒ = 0.25, *α* = 0.05, and 1 − *β* = 0.8 [30].

**Table 1:** The distribution of patients, according to the number of congenitally missing teeth

Number of congenitally missing teeth	Number of patients
1	23
2	21
3	6
4	5
5	1
6	13
7	10
8	8
9	4
10	4
11	4
12	1
13	1
14	1
15	1
16	2
17	0
18	1

### Cephalometric landmarks

**Fig. 1:**
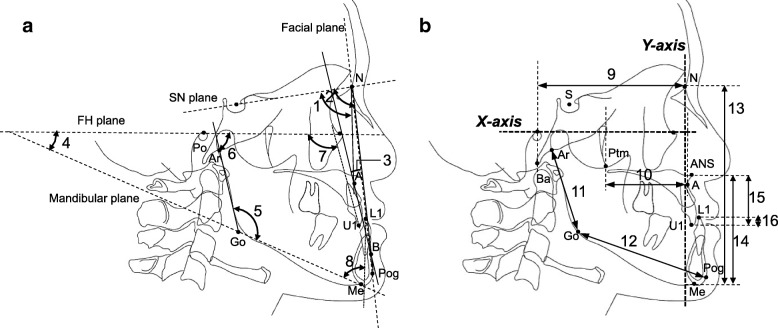
Landmarks and measurements. **a** 1. ∠ SNA, 2. ∠ SNB, 3. ∠ A-B plane angle, 4. ∠ Mandibular plane angle, 5. ∠ Gonial angle, 6. ∠ Ramus inclination, 7. ∠ U1 to FH plane, 8. ∠ L1 to mandibular plane. **b** *X*-axis: parallel to the FH plane, *Y*-axis: perpendicular to the *X*-axis, 9. Ba-N (perpendicular distance to the *Y*-axis), 10. Ptm-A (perpendicular distance to the *Y*-axis), 11. Ar-Go, 12. Go-Pog, 13. N-Me (perpendicular distance to the *X*-axis), 14. ANS-Me (perpendicular distance to the *X*-axis), 15. ANS-U1 (perpendicular distance to the *X*-axis), 16. U1-L1 (perpendicular distance to the *X*-axis)

Lateral cephalograms of the patients were acquired when the teeth were in centric occlusion. Anatomical landmarks were identified on tracing paper. Cephalometric reference points and lines are illustrated in Fig. 1 [31, 32].

### Angular and linear measurements

The cephalometric measurements of eight angles (SNA, SNB, A-B plane angle, mandibular plane angle, gonial angle, ramus inclination, U1 to FH plane, and L1 to mandibular plane) and eight linear parameters (Ba-N, Ptm-A, Ar-Go, Go-Pog, N-Me, ANS-Me, ANS-U1, and U1-L1) were used to evaluate skeletal and dental characteristics. Lengths were measured in accordance with the method described by Coben [33] (Fig. 1). Angular and linear measurements were analyzed by using Z-score values (standard score values) calculated from the Japanese standard norm, as reported by Iizuka et al. and Masaki et al. [31, 32]: Z-score (standard score) = (*X* − *X*′)/*S*′, in which *X* is the patient’s actual measurement, *X*′ is the reference sample measurement in the reference population, and *S*′ is standard deviation of the reference sample in the reference population. All cephalometric tracings were performed by orthodontists (YT).

### Error analysis

Evaluations were performed for both random and systematic errors of the method. To evaluate intra-examiner repeatability, 30 subjects were selected using a random number table—10 from each group—and were retraced 3 weeks later by the same investigator (YT). Additionally, to assess intra-examiner repeatability for the classification of stages by CS, 60 subjects were selected by using a random number table—30 control group and 30 oligodontia group—and then recategorized 3 weeks later by the same investigator (YT). These intra-class correlation coefficient, based on the variance components from one-way analysis of variance, were used. These statistical analyses were performed by using commercially available software (SPSS version 13.0, IBM Corporation, Chicago, IL, USA). The results of the evaluations for random and systematic errors demonstrated excellent intra-examiner agreement: the intra-class correlation coefficient for cephalometric measurements was 0.99; for the classification of stages by CS, it was 0.93.

### Statistical analysis

Kolmogorov-Smirnov analysis indicated that the data were normally distributed (*P* > .05); therefore, parametric tests were used. Intergroup differences among control, hypodontia, and oligodontia groups were analyzed by using one-way analysis of variance and Tukey tests. The *t* test was used to analyze differences between oligodontia and control groups at stage I and stage II. Tukey tests and *t* tests were performed by using STATA SE version 13 (Stata Corp LP, College Station, TX, USA) with statistical significance set at *P* < .05.

## Results

### Cephalometric comparisons of the groups on the basis of the severity of congenitally missing teeth

The mean Z-scores and standard deviation (SD) of individual angles and lengths for control, hypodontia, and oligodontia groups are shown in Table 2. Compared with the control group, the hypodontia group had a significantly smaller U1 to FH plane angle (*P* = 0.002) and A-B plane angle (*P* = 0.038); the U1-L1 (*P* = 0.007) was significantly larger. The oligodontia group had a significantly smaller ANS-Me (*P* = 0.001), L1 to mandibular plane angle (*P* = 0.029), and Ptm-A (*P* = 0.013), and the U1-L1 (*P* = 0.001) was significantly larger. Compared with the hypodontia group, the oligodontia group had a significantly smaller Ptm-A (*P* = 0.007), ANS-Me (*P* = 0.002), SNA (*P* = 0.024), and ANS-U1 (*P* = 0.020), and a significantly larger A-B plane angle (*P* = 0.004).

**Table 2:** Comparisons of cephalometric measurements between control, hypodontia, and oligodontia

	Control (C) (*n* = 63)		Hypodontia (H) (*n* = 56)		Oligodontia (O) (*n* = 50)		C–H	C–O	H–O
	Mean	S.D.	Mean	S.D.	Mean	S.D.	*P* value	*P* value	*P* value
Angular measurements									
SNA	0.00	1.06	0.14	0.83	− 0.36	0.98	0.702	0.129	0.024*
SNB	0.65	1.45	0.58	1.22	0.71	1.65	0.962	0.972	0.885
A-B plane angle	0.98	1.05	0.25	1.68	1.28	2.06	0.038*	0.599	0.004**
Mandibular plane angle	− 0.61	1.45	− 0.40	1.32	− 0.78	1.15	0.661	0.772	0.299
Gonial angle	− 0.07	1.47	− 0.55	2.83	− 0.61	2.26	0.481	0.411	0.988
Ramus inclination	− 0.55	1.17	− 0.36	1.36	− 0.66	1.45	0.732	0.887	0.475
U1 to FH plane	1.75	1.94	0.10	2.63	0.68	3.19	0.002**	0.077	0.486
L1 to mandibular plane	− 0.13	1.20	− 0.45	1.45	− 0.83	1.68	0.454	0.029*	0.360
Linear measurements									
Ba-N	0.11	1.44	0.24	1.58	0.53	1.32	0.877	0.283	0.565
Ptm-A	0.32	1.25	0.38	0.86	− 0.23	0.84	0.944	0.013*	0.007**
Ar-Go	0.80	1.42	0.36	1.50	0.61	1.36	0.220	0.767	0.642
Go-Pog	0.98	1.26	0.54	1.16	0.66	1.15	0.107	0.319	0.865
N-Me	0.64	1.32	0.31	1.36	0.33	1.38	0.420	0.449	1.000
ANS-Me	− 0.22	0.94	− 0.24	1.11	− 0.94	1.07	0.993	0.001**	0.002**
ANS-U1	− 0.98	1.17	− 0.50	1.53	− 1.26	1.64	0.175	0.553	0.020*
U1-L1	− 0.87	1.63	0.21	1.98	0.51	2.17	0.007**	0.001**	0.701

Each number represents the Z-score [(measurement-norm)/SD]. *P < 0.05, **P < 0.01

### Cephalometric comparisons of the oligodontia group on the basis of individual skeletal maturity

The mean Z-scores and SD of individual angles and lengths for oligodontia group and control at either stage I or stage II are shown in Table 3. At stage I, compared with the control group, the oligodontia group had significantly smaller ANS-Me (*P* = 0.001), gonial angle (*P* = 0.020), and ANS-U1 (*P* = 0.025). At stage II, the oligodontia group had a significantly smaller U1 to FH plane angle (*P* < 0.0001), L1 to mandibular plane angle (*P* < 0.0001), Ptm-A (*P* < 0.0001), and Go-Pog (*P* = 0.041), and significantly larger U1-L1 (< 0.0001).

**Table 3:** Comparisons of cephalometric measurements of patients with oligodontia at stage I and stage II

	Stage I (CS2 or 3)					Stage II (CS4 or above)				
	Control (*n* = 31)		Oligodontia (*n* = 27)		*P* value	Control (*n* = 32)		Oligodontia (*n* = 23)		*P* value
	Mean	S.D.	Mean	S.D.		Mean	S.D.	Mean	S.D.	
Angular measurements										
SNA	0.04	0.99	− 0.14	0.79	0.450	− 0.04	1.14	− 0.61	1.13	0.072
SNB	1.10	1.47	1.32	1.51	0.580	0.21	1.30	0.00	1.55	0.583
A-B plane angle	1.63	0.92	1.89	1.92	0.512	0.36	0.75	0.56	2.02	0.594
Mandibular plane angle	− 1.16	1.64	− 1.24	0.99	0.830	− 0.08	1.00	− 0.25	1.11	0.556
Gonial angle	− 0.27	1.72	− 1.54	2.33	0.020*	0.11	1.19	0.48	1.62	0.340
Ramus inclination	− 0.73	1.22	− 0.44	1.44	0.422	− 0.37	1.12	− 0.92	1.46	0.120
U1 to FH plane	2.06	2.41	1.62	3.82	0.595	1.44	1.31	− 0.43	1.74	0.000**
L1 to mandibular plane	− 0.17	1.13	− 0.07	0.97	0.713	− 0.09	1.27	− 1.73	1.90	0.000**
Linear measurements										
Ba-N	0.79	1.29	0.93	1.18	0.671	− 0.55	1.27	0.06	1.35	0.094
Ptm-A	− 0.24	1.47	− 0.39	0.84	0.634	0.87	0.64	− 0.04	0.80	0.000**
Ar-Go	0.99	1.25	0.71	1.19	0.385	0.62	1.57	0.50	1.55	0.784
Go-Pog	0.57	0.95	0.71	0.96	0.586	1.39	1.40	0.60	1.36	0.041*
N-Me	0.83	1.32	0.48	1.39	0.333	0.46	1.32	0.15	1.38	0.411
ANS-Me	− 0.13	1.01	− 1.12	1.08	0.001**	− 0.29	0.87	− 0.72	1.03	0.105
ANS-U1	− 0.69	1.10	− 1.55	1.70	0.025*	− 1.25	1.18	− 0.92	1.54	0.372
U1-L1	− 0.66	1.73	0.10	2.36	0.164	− 1.09	1.53	0.98	1.86	0.000**

Each number represents the Z-score [(measurement-norm)/SD]. *P < 0.05, **P < 0.01

## Discussion

### Influence of congenitally missing teeth on craniofacial morphology

It has been reported that the influence of congenitally missing teeth on craniofacial morphology is strongly associated with the severity of congenitally missing teeth [2, 7, 16, 18–21]. Other authors have reported that craniofacial morphology in individuals with congenitally missing teeth was due to the location of the congenitally missing teeth rather than the severity of congenitally missing teeth [2, 3, 21, 34]. Regarding the influence of congenitally missing teeth severity on craniofacial morphology, patients with congenitally missing teeth exhibited a tendency to more retrognathic maxilla and smaller mandibular plane angle, which led to skeletal class III relationships as the number of congenitally missing teeth increased [16, 18–21]. In this study, the finding of significantly shorter maxillary length and tendency toward small mandibular plane angle in the oligodontia group, compared with the control group, are consistent with findings of previous studies [17, 35]. Despite no significant differences in the A-B plane angle, the mean Z-score of the A-B plane angle for the oligodontia group was + 1.00, which implies that the maxilla was posterior to the mandible, compared with Japanese norms.

Some authors have reported that patients with congenitally missing teeth exhibited more retroclination of the upper incisors [2, 3, 15–17, 19, 21]. They suggested that retroclination of the upper incisors may have been due to reduced lingual support from anterior tooth agenesis. Normally, adjacent teeth prevent lip pressure toward the lingual side; however, no force-resisting lip pressure (from the lack of a lateral incisor) results in retroclination of the upper incisors [3, 16, 17]. In this study, the oligodontia group exhibited a more retrusive lower incisor and no significant differences in the upper incisor compared with the control group, whereas there were significantly more retrusive upper incisors in the hypodontia group than in the control group. Endo et al. [22] reported that anterior tooth agenesis was predominant in mild congenitally missing teeth and that posterior tooth agenesis increased with severity of congenitally missing teeth. We also found that the prevalence of congenitally missing teeth was higher in anterior teeth than in posterior teeth in the hypodontia group (data not shown). Thus, the significant retroclination of the upper incisors in the hypodontia group may be mainly associated with the excessive space created by the lack of anterior teeth. In contrast, retroclination of the lower incisors was found in the oligodontia group. Endo et al. [3] found that retroclination of the lower incisors was more excessive in patients with severe congenitally missing teeth. The authors suggested that this could be a result of compensation for skeletal class III relationships. In the present study, the oligodontia group also had retroclination of the lower incisor, and their skeletal relationships tended to be class III, compared with Japanese norms, which suggests that the findings from our sample may have also been due to dental compensation.

### Comparison of patients with oligodontia on the basis of individual skeletal maturity

The prevalence of non-syndromic oligodontia is ≤ 1% (22); therefore, previous studies investigating craniofacial morphology often have evaluated subjects with a wide range of ages. During alveolar bone development, along with the eruption of permanent teeth, skeletal patterns may differ before and after growth periods in patients with severe congenitally missing teeth. Because it is considered difficult to determine dental age in patients with severe congenitally missing teeth, we investigated the effect of oligodontia on craniofacial morphology according to classifications that used individual skeletal maturity with the CVM method [28].

We observed shorter maxillary anterior facial height and smaller gonial angle, resulting in shorter anterior lower facial height in the oligodontia group, compared with the control group, at stage I. Enlow et al. [23] reported that the vertical growth of the maxillary alveolar bone strongly depends on the vertical drift of the tooth, which implies that patients with congenitally missing teeth are at high risk of losing alveolar height. This hypothesis was supported by Woodworth et al. [35] and Endo et al. [17], in that either bilateral congenital absence of maxillary lateral incisors or congenital absence of ≥ 4 permanent teeth were associated with shorter maxillary alveolar bone height. Furthermore, Mogi et al. [24] reported that the upper first molars were located anteriorly and lower on growing oligodontia patients, which suggests that congenitally missing teeth affect the extent of growth of the maxillary alveolar bone. Previous investigations and the present study suggest that the loss of vertical height is, at least in part, due to the loss of alveolar bone height in patients with congenitally missing teeth. In general, patients with a small gonial angle exhibit shorter facial height [36]. Similar to our results, Nodal et al. [18] reported that patients with severe oligodontia (≥ 13 missing teeth) had a smaller gonial angle, resulting in shorter facial height. Therefore, shorter anterior facial height observed at stage I was likely due to a maxillary alveolar bone growth deficiency and small gonial angle. In contrast, no significant differences were seen at stage II regarding maxillary alveolar bone growth deficiency and small gonial angle, compared with observations at stage I. Although this was not a longitudinal study, the findings suggested that catch-up growth of the maxillary alveolar bone may have occurred. In patients with congenitally missing teeth, the prevalence of congenital absence of the upper second molar is reportedly rare [22], suggesting that the eruption of the upper second molar induces the vertical height. Other factors may include the reason for visiting the hospital in patients with oligodontia because patients at stage II mostly visited our hospital for treatments to close or maintain spaces that occurred due to congenitally missing teeth (i.e., for dental prosthesis). Many patients thought that there was no skeletal problem until the end of the growth period and did not require orthodontic treatment. In contrast, at stage I, patients visited for treatment to close or maintain spaces and to treat skeletal class III deficiency, which may have been the reason these unique features were detected among the groups.

In this study, the patients with oligodontia exhibited retroclination of the upper and lower incisors and increased overbite at stage II. Some authors have reported that retroclination of upper and lower incisors was more excessive in patients with severe congenitally missing teeth [2, 17]. They suggested that this could be due to space created by the lack of congenitally missing teeth or compensation for the skeletal class III relationships. The common knowledge regarding the persistence of primary teeth is that primary teeth may be retained because of the absence of the permanent successor, and less resorption of primary tooth roots has been encountered [37]. However, persistent primary teeth may exfoliate soon due to growth. The retroclination of upper and lower incisors could be due to the excessive space created by the lack of persistence of primary teeth in patients with oligodontia at stage II.

When considering treatment plans for stage I oligodontia patients, the study, although retrospective, implies that anterior vertical height may have been improved by growth modification; thus, rather than planning early treatment, clinicians should consider waiting until patients’ growth has progressed. However, decisions regarding early treatment depend on the severity of shorter vertical height and other abnormalities of craniofacial morphology.

### Limitations

The main limitation of the present study was its retrospective design. All samples, including controls, were collected from Japanese orthodontic patients, which limit the generalizability of our study; specifically, because samples were taken only from a Japanese population, the results may not be applicable to other ethnic groups.

## Conclusions

The present study suggested that skeletal patterns differ with the number of congenitally missing teeth and that, in oligodontia patients, skeletal patterns differ before and after growth peak. Thus, when designing orthodontic treatment plans for those with congenitally missing teeth, orthodontists should consider that craniofacial morphology may be altered because of variations associated with severity of congenitally missing teeth and because of individual skeletal maturity.

## Abbreviations

CS: Cervical stage
CVM: Cervical vertebral maturation

## References

[1] Khalaf K, Miskelly J, Voge E, Macfarlane TV (2014). Prevalence of hypodontia and associated factors: a systematic review and meta-analysis. J Orthod.

[2] Ben-Bassat Y, Brin I (2003). Skeletodental patterns in patients with multiple congenitally missing teeth. Am J Orthod Dentofac Orthop.

[3] Endo T, Ozoe R, Yoshino S, Shimooka S (2006). Hypodontia patterns and variations in craniofacial morphology in Japanese orthodontic patients. Angle Orthod.

[4] Sisman Y, Uysal T, Gelgor IE (2007). Hypodontia. Does the prevalence and distribution pattern differ in orthodontic patients?. Eur J Dent.

[5] Rakhshan V (2013). Meta-analysis and systematic review of factors biasing the observed prevalence of congenitally missing teeth in permanent dentition excluding third molars. Prog Orthod.

[6] Gracco ALT, Zanatta S, Forin Valvecchi F, Bignotti D, Perri A, Baciliero F (2017). Prevalence of dental agenesis in a sample of Italian orthodontic patients: an epidemiological study. Prog Orthod.

[7] Bondarets N, McDonald F (2000). Analysis of the vertical facial form in patients with severe hypodontia. Am J Phys Anthropol.

[8] Bailleul-Forestier I, Berdal A, Vinckier F, de Ravel T, Fryns JP, Verloes A (2008). The genetic basis of inherited anomalies of the teeth. Part 2: syndromes with significant dental involvement. Eur J Med Genet.

[9] Jamilian A, Lucchese A, Darnahal A, Kamali Z, Perillo L (2016). Cleft sidedness and congenitally missing teeth in patients with cleft lip and palate patients. Prog Orthod.

[10] Schalk-van der Weide Y, Steen WH, Bosman F (1992). Distribution of missing teeth and tooth morphology in patients with oligodontia. ASDC J Dent Child.

[11] Stockton DW, Das P, Goldenberg M, D'Souza RN, Patel PI (2000). Mutation of PAX9 is associated with oligodontia. Nat Genet.

[12] Higashihori N, Takada J, Katayanagi M, Takahashi Y, Moriyama K. Frequency of missing teeth and reduction of mesiodistal tooth width in Japanese patients with tooth agenesis. Prog Orthod. In press 10.1186/s40510-018-0222-4 PMC6098995 30123921

[13] Wisth PJ, Thunold K, Böe OE (1974). The craniofacial morphology of individuals with hypodontia. Acta Odontol Scand.

[14] Roald KL, Wisth PJ, Bøe OE (1982). Changes in cranio-facial morphology of individuals with hypodontia between the ages of 9 and 16. Acta Odontol Scand.

[15] Sarnäs KV, Rune B (1983). The facial profile in advanced hypodontia: a mixed longitudinal study of 141 children. Eur J Orthod.

[16] Ogaard B, Krogstad O (1995). Craniofacial structure and soft tissue profile in patients with severe hypodontia. Am J Orthod Dentofac Orthop.

[17] Endo T, Yoshino S, Ozoe R, Kojima K, Shimooka S (2004). Association of advanced hypodontia and craniofacial morphology in Japanese orthodontic patients. Odontology.

[18] Nodal M, Kjaer I, Solow B (1994). Craniofacial morphology in patients with multiple congenitally missing permanent teeth. Eur J Orthod.

[19] Ben-Bassat Y, Brin I (2009). Skeletal and dental patterns in patients with severe congenital absence of teeth. Am J Orthod Dentofac Orthop.

[20] Chan DW, Samman N, McMillan AS (2009). Craniofacial profile in Southern Chinese with hypodontia. Eur J Orthod.

[21] Gungor AY, Turkkahraman H (2013). Effects of severity and location of nonsyndromic hypodontia on craniofacial morphology. Angle Orthod.

[22] Endo T, Ozoe R, Kubota M, Akiyama M, Shimooka S (2006). A survey of hypodontia in Japanese orthodontic patients. Am J Orthod Dentofac Orthop.

[23] Enlow DH, John D (1990). The facial growth process: physiologic tooth movements and alveolar remodeling. Facial growth.

[24] Mogi K, Ogawa T, Baba Y, Moriyama K (2012). Craniofacial morphology in growing patients with oligodontia. Orthod Waves Jpn Ed.

[25] Badrov J, Lauc T, Nakaš E, Galić I (2017). Dental age and tooth development in orthodontic patients with agenesis of permanent teeth. Biomed Res Int.

[26] Franchi L, Baccetti T, McNamara JA (2000). Mandibular growth as related to cervical vertebral maturation and body height. Am J Orthod Dentofac Orthop.

[27] Baccetti T, Franchi L, McNamara JA (2002). An improved version of the cervical vertebral maturation (CVM) method for the assessment of mandibular growth. Angle Orthod.

[28] McNamara JA, Franchi L (2018). The cervical vertebral maturation method: a user’s guide. Angle Orthod.

[29] Cohen J (1992). A power primer. Psychol Bull.

[30] Faul F, Erdfelder E, Buchner A, Lang AG (2009). Statistical power analyses using G*Power 3.1: tests for correlation and regression analyses. Behav Res Methods.

[31] Iizuka T (1958). Roentgencephalometric analysis of craniofacial growth in Japanese children. J Stomatol Soc Jpn.

[32] Masaki F (1980). The longitudinal study of morphological differences in the cranial base and facial structure between Japanese and American whites. Orthod Waves Jpn Ed.

[33] Coben SE (1955). The integration of facial skeletal variants: a serial cephalometric roentgenographic analysis of craniofacial form and growth. Am J Orthod.

[34] Yüksel S, Uçem T (1997). The effect of tooth agenesis on dentofacial structures. Eur J Orthod.

[35] Woodworth DA, Sinclair PM, Alexander RG (1985). Bilateral congenital absence of maxillary lateral incisors: a craniofacial and dental cast analysis. Am J Orthod.

[36] Proffit WR, Sarver DM, Ackerman JL, Proffit WR, Fields HW, Sarver DM (2013). Orthodontic diagnosis: the problem-oriented approach. Contemporary orthodontics.

[37] Aktan AM, Kara I, Sener I, Bereket C, Celik S, Kirtay M (2012). An evaluation of factors associated with persistent primary teeth. Eur J Orthod.

